# Innate immunity to malaria: The good, the bad and the unknown

**DOI:** 10.3389/fimmu.2022.914598

**Published:** 2022-08-19

**Authors:** Kai Pohl, Ian A. Cockburn

**Affiliations:** ^1^ Department of Infectious Diseases and Respiratory Medicine, Charité - Universitätsmedizin Berlin, Freie Universität Berlin and Humboldt-Universität Berlin, Berlin, Germany; ^2^ Division of Immunology and Infectious Disease, John Curtin School of Medical Research, The Australian National University Canberra, Canberra, ACT, Australia

**Keywords:** malaria, sporozoites, innate immunity, blood stages, pattern recognition receptors, pathogen-associated molecular patterns, *Plasmodium*

## Abstract

Malaria is the cause of 600.000 deaths annually. However, these deaths represent only a tiny fraction of total malaria cases. Repeated natural infections with the causative agent, *Plasmodium* sp. parasites, induce protection from severe disease but not sterile immunity. Thus, immunity to *Plasmodium* is incomplete. Conversely, immunization with attenuated sporozoite stage parasites can induce sterile immunity albeit after multiple vaccinations. These different outcomes are likely to be influenced strongly by the innate immune response to different stages of the parasite lifecycle. Even small numbers of sporozoites can induce a robust proinflammatory type I interferon response, which is believed to be driven by the sensing of parasite RNA. Moreover, induction of innate like gamma-delta cells contributes to the development of adaptive immune responses. Conversely, while blood stage parasites can induce a strong proinflammatory response, regulatory mechanisms are also triggered. In agreement with this, intact parasites are relatively weakly sensed by innate immune cells, but isolated parasite molecules, notably DNA and RNA can induce strong responses. Thus, the innate response to *Plasmodium* parasite likely represents a trade-off between strong pro-inflammatory responses that may potentiate immunity and regulatory processes that protect the host from cytokine storms that can induce life threatening illness.

## Introduction

Malaria remains a major cause of morbidity and mortality worldwide with an estimated 627,000 deaths in 2020 (1), though this represents a small fraction of the over 200 million clinical cases per year. Moreover, in high transmission areas, 40-70% of individuals can harbor malaria parasites in their blood as asymptomatic infections (2). Thus, while the overall burden of disease is high, case fatality is low. Moreover, even though asymptomatic individuals have lower levels of infection than individuals with severe or mild infection, immunity does not control infection and parasite biomass is high. However, in semi-immune individuals this parasitaemia does not induce the florid and life-threatening innate response associated with bacteraemia and sepsis. Thus, a key outstanding question is how malaria infections can be tolerated by the immune system, while also being controlled. A further outstanding question is that while blood stages appear to be largely tolerated by the immune system, other lifecycle stages - notably the sporozoite and liver stages are rapidly sensed by the innate response and can induce robust immune responses.

Human malaria is caused by one of five species of the single-celled eukaryotic genus *Plasmodium;* with the highest disease burden caused by *P. falciparum (* 3, 4). *Plasmodium* parasites have a complex lifestyle that entails sexual reproduction in the *Anopheles* vector and asexual reproduction in the human host [reviewed elsewhere (4, 5)]. Human infection begins when sporozoites are delivered into the skin by the bites of an infected *Anopheles* mosquito. Sporozoites travel from the skin *via* the bloodstream to the liver, where they invade hepatocytes. During the liver stages, a single parasite gives rise to tens of thousands of merozoites which are then released into the bloodstream initiating the symptomatic blood stage of a *Plasmodium* infection by replicating inside of erythrocytes. Importantly, while only tens to hundreds of sporozoites are injected into the dermis per mosquito bite, one successfully developing sporozoite is enough to cause blood stage infection with an average of 7 x 10^11^ blood-borne parasites (6). A subset of these blood stages will develop into sexual stage gametocytes which can continue the cycle of infection if taken up during blood feeding by another mosquito (5).

Symptoms of malaria range from none in partially immune individuals, to cyclic fever and to severe manifestations leading to death. However only around 1% of clinical infections lead to severe malaria, most often in immunological naïve individuals (3). Accordingly, in endemic areas children under 5 years of age are most affected and most likely to suffer from severe malaria and death (3). Semi-immunity, characterized by the host’s ability to tolerate and - to an extent - suppress blood stage infection, is acquired over the course of several disease episodes (7, 8). This semi-immunity is most often attributed to antibody-mediated control of parasitemia (resistance) (7) and to an ability to tone down overarching inflammatory responses during peak infection (tolerance) (8). Importantly, even numerous blood stage infections do not induce sterile immunity and people living in endemic areas are repeatedly infected throughout their lives. This fact has led to the notion that blood stage infection induces a form of unnatural and ineffective immunity.

While blood stage infection induces strong inflammatory responses in naïve individuals but not sterile immunity, it has long been known that attenuated sporozoites can induce sterile immunity, which has been a major model for vaccination. This is true whether sporozoites are attenuated by irradiation, drug control of subsequent blood stage infection, or knockout of key genes required for progression from liver stages to blood stages (9–11). However, sporozoite vaccines are hampered by the logistical challenges associated with the preparation of large amounts of sporozoites, the possibility of breakthrough infections and reduced efficacy in endemic settings (12, 13). Nonetheless, compared to blood stage infection - relatively small numbers of sporozoites induce a strong adaptive immunity characterized by high titers of affinity matured antibodies and strong CD8 T-cell responses (14). In this regard, an outstanding question in the field has been how different life cycle stages of the same parasites induce such different adaptive immune responses.

## Innate immunity to pre-erythrocytic stages

While sporozoites are evolutionarily optimized for their journey to the liver, only a few manage to infect hepatocytes with the remaining sporozoites being cleared by cells of the innate immune system. These unsuccessful sporozoites likely contribute to innate immune activation and are a source of antigen for T and B cells (15). *In vivo* tracking of fluorescent sporozoites after intradermal injection revealed that similar numbers of viable *P. berghei* sporozoites reached the liver or remained in the skin (16). Importantly, sporozoites were found in subcapsular zones of skin draining lymph nodes (dLNs) before being taken up into dLN resident CD8^+^ Dendritic cells (DCs) which efficiently prime CD8^+^ T-cells (16, 15),. The important role for mouse CD8^+^ cDC1s in priming sporozoite-specific CD8^+^ T-cells was also shown in Batf3^-/-^ mice which lack cDC1s (15, 17, 18). In addition, targeted expression profiling of dLNs 24 h after sporozoite injection revealed elevated expression of CXCL9, CXCL10, Granzyme B and IFNγ (16). Work done with the *P. yoelii* model further suggested that priming in dLNs after subcutaneous sporozoite injection is sufficient to induce protective immunity (19, 20). IFNγ, dendritic cells as well as CD4^+^ and CD8^+^ T-cells also play important roles during the induction of immunity, while CD8^+^ T-cells were most important for protection from challenge later on (20).

Beyond animal models, controlled human malaria infections, often as part of clinical trials of whole sporozoite vaccines have also provided insights into protective immune responses. Interestingly, one of the strongest correlates of protection in sporozoite vaccinated individuals was increased frequencies of Vγ9^+^ Vδ2^+^ T-cells at baseline and 2 weeks after final immunization (14). In line with these findings, RNA sequencing of PBMCs isolated from vaccinated individuals identified that elevated expression of two genes encoding γδTCRs (TRDV2 and TRGV9) at 3 days after the last vaccination were associated with protection from mosquito-bite challenge (18). Gamma-delta T cells are an innate-like population of T-cells that express a limited repertoire of γδTCRs. Only very few ligands for γδTCRs have been identified so far (21), however, even without vaccination, γδT-cells in *in vitro Pf*SPZ-stimulated PBMCs produced IFNγ, indicating the presence of a γδTCR ligand in sporozoites (14). In mouse models, depletion of γδT-cells during sporozoite vaccination ablated protective immunity mediated by CD8+ T cells further supporting a role for this population in the induction of adaptive immune responses (18).

**Figure 1:**
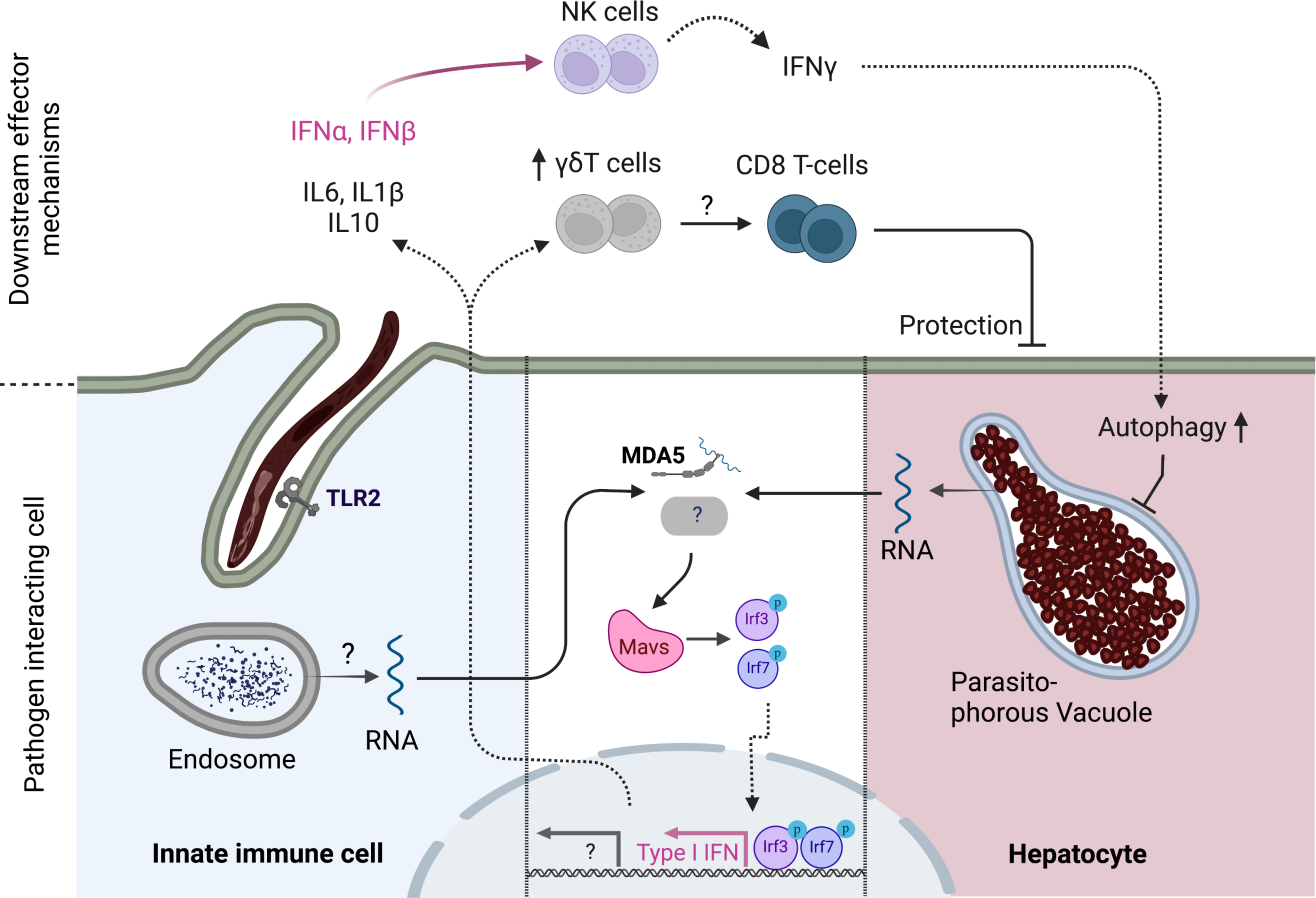
Innate immune response to pre-erythrocytic stages of *Plasmodium* parasites. Cytoplasmic *Plasmodium* RNA is sensed by MDA5 which signals *via* MAVS. MAVS activation ultimately leads to the phosphorylation of transcription factors IRF3 and IRF7 which drive the expression of Type I IFN genes such as IFNα and IFNβ. IFNα,β recruit NK cells to the liver which produce IFNγ which in turn increases autophagy pathways in hepatocytes. Another, yet unidentified, PRR might signal *via* MAVS to enhance this response. The cell type of origin of the initial Type I IFN response has not been identified and could either be infected hepatocytes or tissue resident innate cells that have taken up parasite material. TLR2 has been shown to be capable of sensing sporozoites, leading to reduced liver stage burden and enhanced inflammatory gene expression in mice. In addition, γδT-cells have been linked to favorable vaccination outcomes in humans and play a role in inducing a protective CD8 T-cell response in concert with CD8^+^ cDC1s in mice. Created with Biorender.

Once in the liver, intrahepatic stages of the rodent *P. berghei* parasite induce the expression of type I IFNs (22). This response appeared dependent on parasite replication, as irradiated parasites induced markedly reduced levels of IFN expression (22). This response was abrogated in mice deficient for Mitochondrial antiviral-signaling protein (MAVS) (22). MAVS is the adaptor protein for the cytosolic RNA sensors Melanoma differentiation-associated protein 5 (MDA5) and Ritionic acid inducible gene I (RIG-I) (23). Upon activation, for example during viral infection, MAVS induces the expression of Type I IFN and thus contributes to cell intrinsic and extrinsic defense against pathogens (23). Interestingly, cytokine production in response to intrahepatic parasites was only partially reduced in MDA5 deficient mice and not affected in Rig-I deficient animals, suggesting the existence of another cytosolic pattern recognition receptor (PRR) capable of detecting parasite pathogen associated molecular patterns (PAMPs). The cell type that senses *Plasmodium* RNA was not identified and could be either innate immune cells or the hepatocyte itself (**Figure 1**) (22). Simultaneously, a second group suggested that type I IFN may recruit IFN-gamma producing NK cells to the liver to reduce hepatic parasite burden (24). A possible mechanism through which IFNγ can mediate a reduction of liver stage burden could be an autophagy pathway that kills intrahepatic parasites (25, 26)

*In vivo* experiments can be complemented by *in vitro* co-culture experiments of sporozoites with either primary or cultured innate immune cells. However, such studies are hampered by difficulties obtaining pure populations of sporozoites. Initial experiments co-culturing *P. yoelii* sporozoites with mouse peritoneal macrophages revealed that sporozoites induced a respiratory burst in macrophages (27). Interestingly, frequencies of activated macrophages were lower when using salivary gland sporozoites than when immature oocyst-like sporozoites were used (27). Shortly after, Vanderberg and colleagues (28) characterized sporozoite macrophage interactions *in vitro* using live cell imaging and discovered multiple modes of host-parasite interaction. Importantly, sporozoites were shown to actively penetrate and subsequently egress from macrophages, a process that in some cases led to the destruction of the innate cells. This traversal process has subsequently been shown to be of great importance for sporozoites *in vivo* to leave the dermis after deposition through a mosquito bite and traverse endothelial cells and Kupffer cells prior to infecting hepatocytes (29–31). Host-cell traversal also induces the loss of macrophage plasma membrane integrity (32) and activates Kupffer cells (33).

In addition to cytosolic RNA sensors identified *in vivo*, Toll-like receptor 2 (TLR2) has also been identified as an innate sensor of sporozoite infection from *in vitro* studies (34). TLR2 belongs to the Toll like receptor family whose members can sense a range of PAMPs in the extracellular space or the endosome (35). Most TLRs signal *via* MyD88 to induce strong NF-κB driven pro-inflammatory gene expression and the secretion of cytokines such as IL6 and TNFα (35). It was further shown that TLR2 deficient mice had higher liver stage burden after intravenous sporozoite injection and failed to express inflammatory cytokines in the liver. However it is not clear whether TLR2 acts in concert with MDA5 and MAVS to induce a hepatic type I IFN response (34). Another recent *in vitro* study characterized human innate cell co-culture with *P. berghei* and *P. falciparum* sporozoites, showing that the parasites were readily taken up into monocyte derived macrophages and DCs, which in turn produced IL-6, IL-1β and IL10 (36). Overall, sporozoites appear to be potent inducers of pro-inflammatory immune responses that potentiate adaptive immunity, however, both the interacting cells and the ligand-receptor pairs that mediate these responses remain poorly characterized.

## Innate immunity to blood stage infection

While sporozoites appear to be potent stimulators of both innate and adaptive immune responses especially when considered on a per parasite basis, the pre-erythrocytic stages of malaria infection are clinically silent because the number of invading parasites is so small. In contrast, blood stage infection is responsible for the disease manifestations of malaria which can be characterized by sepsis-like excessive inflammation in naïve individuals (3, 37). However, many immunopathology mechanisms during severe blood stage infection target specific tissues and rely on tissue-specific host-parasite interactions (38). Key virulence factors for malaria include various families of antigenically variant surface antigens (VSAs), including the var (encoding PfEMP1), rif (encoding RIFIN) and stevor (encoding STEVOR) gene families of *P. falciparum* that encode around 60, 200 and 30 highly polymorphic genes, respectively (39–41). In particular, the PfEMP1 family of surface receptors allow infected erythrocytes to adhere to endothelial cells allowing for the sequestration to prevent their removal *via* the spleen. These adhesion processes are associated with severe disease (42), in particular cerebral malaria due to obstruction of blood vessels in the brain. In addition to var genes, RIFINs also play roles in immune modulation. Three human receptors have been found to bind to *P. falciparum* RIFINs; LAIR1 (43, 44), LILRB1 (45, 46) and LILRB2 (47). All of these receptors belong to the Ig superfamily, contain intracellular immunoreceptor tyrosine-based inhibitory (ITIM) motifs and are broadly expressed by myeloid cells and lymphocytes (48–50). ITIM motifs downregulate cell activation by antagonizing activation signals in many different immunological contexts, such as NK-cell activation, T and B cell activation and myeloid cell activation after PRR engagement (51).

Seminal work showed that PfEMP1 can also be used by infected erythrocytes to bind to human DCs *via* CD36 resulting in the inhibition of LPS-induced maturation and a reduced ability of DCs to induce T-cell proliferation (52). In human monocytes, *Pf*EMP1 expressing parasites also induced weaker inflammatory cytokine expression, corroborating the immune modulating role of *Pf*EMP1 *(* 53). Interestingly, More recent work characterizing human dendritic cell responses to blood stage parasites also found atypical activation patterns with a marked absence of inflammatory cytokine production and low co-stimulatory molecule expression (54). Nonetheless, DCs stimulated with intact parasitized erythrocytes were able to potently activate CD4^+^ T-cells *in vitro* marked by the induction of high levels of IFNγ and TNFα (54). A similar activation phenotype was also observed in DCs isolated from individuals from endemic countries (54, 55).

Rodent malaria models have also provided key insights into innate immune interactions with blood stage parasites, circumventing some of the limitations of *in vitro* studies that use long-term cultured *P. falciparum* strains. In agreement with human data, mouse bone marrow derived DCs also show reduced maturation capacity upon stimulation with LPS, when they were pre-treated with *P. yoelii* blood stage parasites *ex vivo (* 56). In addition, it was shown that DCs stimulated with *ex vivo* purified *P. yoelii* infected erythrocytes produced soluble mediators that reduced CD8^+^ T-cell activation, giving rise to the idea that blood stage infection could suppress immune responses to pre-erythrocytic stages. Later work, however, found no defect in infected erythrocyte-induced DC maturation (57), and confirmed the ability of DCs to phagocytose parasites and induce long lasting immune responses (58). In agreement with this, *in vivo* studies in the *P. berghei* ANKA model, which is characterized by T-cell dependent cerebral cytotoxicity (59), have shown that depletion of conventional DCs ameliorated T-cell mediated immune pathology, while parasite control remained unaffected (60, 61). These contrasting findings highlight that DC biology during blood stage malaria is still incompletely understood and important differences are present between rodent and human host-pathogen pairs likely determined by differences in the VSAs on infected erythrocytes.

**Figure 2:**
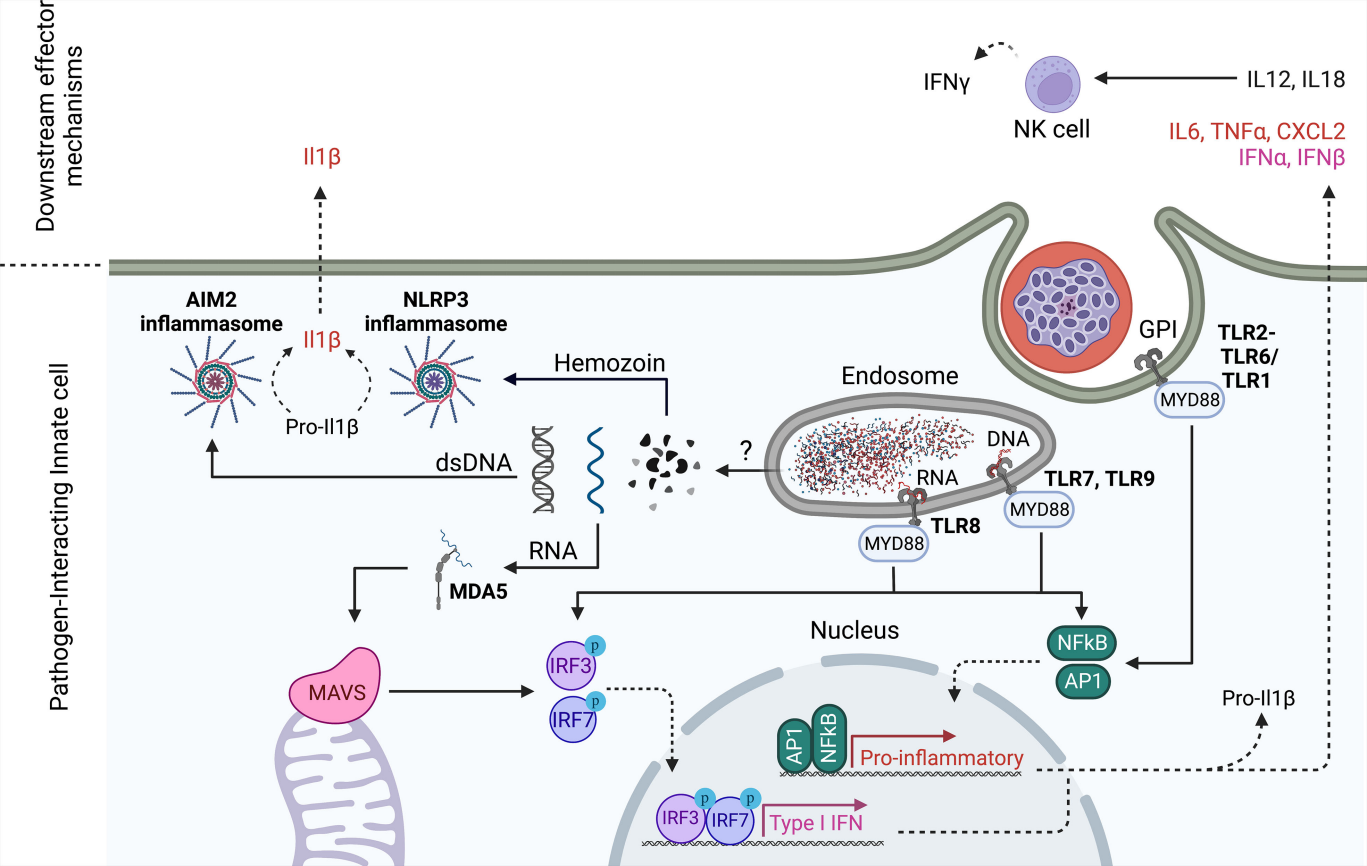
Innate sensing mechanisms of *Plasmodium* blood stages. TLR2 senses *Plasmodium* GPIs on the cell surface, while TLR7 and 9 recognize *Plasmodium* DNA in the endosome. In humans, TLR8 also senses degradation products of *Plasmodium* RNA. TLR engagement drives pro-inflammatory gene expression and a Type I IFN response (only TLR7, 8,9) that is dependent on MyD88 signaling. *Plasmodium* nucleic acids escape from the lysosome probably through direct association with hemozoin. In analogy to pre-erythrocytic stages, *Plasmodium* RNA in the cytoplasm is detected by MDA5 leading to MAVS activation and downstream Type I IFN gene expression. In addition, cytoplasmic double stranded (ds)DNA is sensed by AIM2 while Hemozoin is sensed by NLRP3, each leading to inflammasome assembly and enzymatic cleavage of pro-inflammatory mediators like Pro-IL1β. Akin to pre-erythorcytic stages, NK cells have been shown to produce IFNγ downstream of PRR recognition of parasite PAMPs. In *ex vivo* culture systems of human cells, NK cells have been shown to produce IFNγ in response to innate cell produced IL18 and IL12 which were dependent on TLR8. Created with Biorender.

In addition to understanding cellular host-parasite interactions, research into innate immunity to blood stages has focused on identifying parasite immune stimulatory ligands and their host cell receptors (**Figure 2**). The first candidate parasite PAMPs identified were Glycosylphosphatidylinositols (GPIs), which are complex lipid, carbohydrate and phosphate containing molecules that are common in all eukaryotic life and function to anchor proteins to membranes (62). *Plasmodium* GPIs contain conserved molecular features that are distinct from human GPIs (62). Early studies identified *P. falciparum* GPIs as immune stimulatory ligands for innate cells that induced the production of TNFα and IL-1β in macrophages and resulted in immune pathology in mice upon injection (63). TLR2 was later identified to be the receptor for GPIs of several protozoan parasites including plasmodia through the formation of heterodimers with either TLR1 or TLR6 (64, 65).

A second well-researched blood stage PAMP is hemozoin. Through digestion of haemoglobin, intraerythrocytic *Plasmodium* parasites generate toxic heme, which can produce free radicals in the parasite digestive vacuole. To detoxify heme, *Plasmodium* parasites convert it into an insoluble crystallized form, called hemozoin which can be released into the bloodstream during rupture of the infected red blood cell (66). Hemozoin has been studied extensively in its ability to induce innate activation and has led to many confusing findings mainly due to its ability to complex many biological entities including lipids and nucleic acids that themselves can activate PRRs (67, 68). The study of synthetic hemozoin that is free of contaminants in *in vitro* systems has ultimately led to the idea that hemozoin itself is relatively inert to innate cells, but functions by delivering PRR ligands to their respective receptors (69). However, there are also reports that challenge this notion and attribute the release of numerous inflammatory cytokines such as IL6 and IL1ß to the sensing of hemozoin through the NLRP3 inflammasome in mice (70–72). Inflammasomes are multi-protein complexes that assemble in response to environmental triggers in the host cell cytosol. Once assembled, these complexes enzymatically cleave pro-forms of the highly inflammatory cytokines IL-1ß and IL18 into their respective bioactive forms (73). Inflammasomes thus play critical roles in pathogen defense but have also been implicated in autoimmune disorders (73).

The investigation of hemozoin as a malaria PAMP is tightly connected to the study of plasmodial DNA as activating ligand of innate immune cells. As such, it was first thought that hemozoin is the activating ligand of TLR9 (74, 75). However, subsequent studies in which hemozoin was prepared free of DNA contamination and used to stimulate mouse bone marrow derived DCs showed that in fact *Plasmodium* DNA was the TLR9 ligand and that it was bound to hemozoin (69). The ability of human TLR9 to sense plasmodial DNA has been confirmed by independent groups (76, 77). Interestingly, hemozoin seems to have an adjuvating effect when it is complexed with DNA (69), supposedly by allowing DNA access to the cytosol where it also activates cytosolic DNA sensors such as AIM2 (78). However, it is still unclear how hemozoin escapes from endosomes. Upon ligand engagement, endosomal DNA sensors TLR7 and TLR9 predominantly initiate a type I IFN response through the activation of IRF transcription factors. In addition, type I IFN production was also shown to be induced after cGAS-mediated detection of *Plasmodium* DNA in the cytoplasm (79). Again, the authors show that DNA access to the cytosol is mediated by complex formation with hemozoin (79).

RNA sensing mechanisms and their role during disease have also been studied. While in mice, MDA5 seems to be activated during blood stage *P. yoelii* infection to signal *via* MAVS the production of type I IFNs (80), the activating ligand for MDA5 has not clearly been identified yet. Interestingly, the authors found that ablating IFN production after cytosolic DNA or RNA sensing protected mice from lethal *P. yoelii* challenge, while abrogating endosomal nucleic acid sensing through TLR7 and TLR9 did not (80). More recently, Coch et al (81) discovered that human TLR8 senses *Plasmodium* RNA in the endosome leading to robust induction of IL-1β in the human THP-1 cell line that was abrogated in TLR8 deficient THP-1 cells. Shortly after, RNAse T2 was shown to degrade endosomal *Pf*RNA prior to sensing of degradation products by TLR8 (82). When human PBMCs were stimulated with blood stage parasites, TLR8 contributed to mounting a partly NK-cell dependent IFNγ response (81), which is in line with earlier findings that the TLR8 adaptor MyD88 is necessary for *P. falciparum* blood stage induced IFNγ production by NK cells (83).

## Concluding remarks

Blood stage malaria manifests as a severe inflammatory disease in naïve individuals. In animal models, genetic ablation of innate sensing pathways often offer survival benefits pointing towards a role for dysregulated inflammatory signaling in innate cells in malaria pathology (80). However, reductionist co-culture systems with intact parasites generally seem to reveal nuanced responses of innate cells that often show a lack of, or reduced inflammatory signaling (54), unless parasites are added in high quantities or stimulatory ligands are purified.

To understand this seeming contradiction, a biomass comparison may be made between a *Plasmodium* blood stage infection, sporozoite immunization and sepsis caused by *E. coli* (**Table 1**). A blood stage *Plasmodium* infection can involve hundreds of billions of parasites with a total biomass of several grams. Nonetheless, such infections are frequently tolerated by the host, even in naive or semi-immune individuals. In contrast, a septicemic *E. coli* infection can induce a life-threatening cytokine storm with one millionth of the amount of antigen. This comparison argues that on a per-pathogen and per gram biomass basis, blood stage parasites have a relatively low inflammatory capacity as compared to bacteria. Interestingly, comparatively low numbers of sporozoites can induce sterile immunity when used as a vaccine, while 6 orders of magnitude more blood stage parasites do not suffice.

**Table 1:** Comparison of pathogen numbers and biomass during *P. falciparum* blood stage infection, sporozoite vaccination and *E. coli* mediated sepsis.

	Blood stage infection	Sporozoite vaccine	E. coli sepsis
Total number of pathogens in blood	7 x 10^11^ [ref (6)]	2 x 10^5^ [ref (84)]	4 x 10^6^ [ref (85)]
Weight per pathogen	34.6 pg [ref (86)]	34.6 pg [ref (86)]	5 pg [ref (87)]
Total pathogen biomass	24.200.000 μg	6.92 μg	19.5 μg

Pathogen numbers were curated and calculated from references indicated in the table for an average human female. Weight for average trophozoites was approximated using volumetric measurements from indicated reference (49 fl) and the dry weight to water ratio of bacteria (0.22). In rough approximation, the same weight was assumed for sporozoites.

Understanding the mechanistic basis for this difference could be highly valuable. An explanation for these observations can likely be found considering the pressure that evolution exerts on host parasite interactions. This pressure likely favored different outcomes for pre-erythrocytic and blood stage parasites, especially considering their respective roles during the parasite life cycle: While only a single sporozoite needs to productively infect a hepatocyte to complete its mission, blood stage parasites need to keep proliferating in the blood for long periods of time to ensure successful uptake of gametocytes into a feeding mosquito.

Thus, blood stage parasites were subject to high evolutionary pressure to survive host sensing pathways to avoid destruction by innate or adaptive immunity. On the other hand, sporozoites might have evolved to very efficiently reach host hepatocytes while being much less manipulative regarding innate sensing pathways. Their high success rate allows only very few sporozoites to be deposited into the skin during a mosquito blood feed, limiting the amount of PAMPs to be detected and the amount of antigen available for the induction of protective immunity.

Ultimately, a deeper understanding of innate pathways that are activated by the parasite’s life cycle stages and their respective downstream contributions towards beneficial *vs*. detrimental inflammatory and adaptive immune responses will be needed to guide both treatment and prevention strategies of the future.

## Author contributions

KP drafted the original manuscript, prepared the figures and redrafted the manuscript. IC reviewed, redrafted and edited the manuscript for submission. All authors contributed to the article and approved the submitted version.

## Funding

KP was supported by PhD studentship from the Deutsche Forschungsmeinschaft Crossing Boundaries Molecular Interactions in Malaria International Research Training Group Program (IRTG2290).

## Conflict of interest

The authors declare that the research was conducted in the absence of any commercial or financial relationships that could be construed as a potential conflict of interest.

## Publisher’s note

All claims expressed in this article are solely those of the authors and do not necessarily represent those of their affiliated organizations, or those of the publisher, the editors and the reviewers. Any product that may be evaluated in this article, or claim that may be made by its manufacturer, is not guaranteed or endorsed by the publisher.
